# Surgical and irradiated case of early breast cancer in a patient with Ehlers–Danlos syndrome

**DOI:** 10.1186/s40792-024-01997-5

**Published:** 2024-08-23

**Authors:** Asumi Yamazaki, Hiroshi Tada, Yuki Muroyama, Yuto Yamazaki, Minoru Miyashita, Narumi Harada-Shoji, Yohei Hamanaka, Akiko Ebata, Miku Sato, Tokiwa Motonari, Mika Yanagaki, Tomomi Kon, Aru Sakamoto, Takashi Suzuki, Takanori Ishida

**Affiliations:** 1 Department of Breast and Endocrine Surgical Oncology, Graduate School of Medicine, Tohoku University, Sendai, Japan; 2 Department of Pathology, Tohoku University Hospital, Sendai, Japan

**Keywords:** Ehlers–Danlos syndrome, Breast cancer, Predict, BluePrint

## Abstract

**Background:**

Ehlers–Danlos syndrome (EDS) is a rare inherited connective tissue disease characterized by hyperextensibility of the skin and joints and tissue fragility of the skin and blood vessels, Vascular EDS is the most severe form of EDS, with abnormal arterial fragility. There have been no reports of breast cancer occurring in patients with vascular EDS. Here, we report here a very rare case of breast cancer in a patient with vascular EDS.

**Case presentation:**

A 46-year-old woman with vascular EDS underwent partial left mastectomy and sentinel lymph node biopsy for left breast cancer (cStage 0) detected by medical examination. The final pathological diagnosis was invasive ductal carcinoma of the breast (pStage IA) [hormone receptor-positive, HER2 score 2 equivocal (FISH-positive), Ki-67LI 18%, luminal-HER2 type]. BluePrint was submitted as an aid in determining the postoperative treatment strategy, BluePrint Molecular Subtype HER2-type. However, the 10-year breast cancer mortality risk using Predict was low (5%). After consultation with the patient, the decision was made to administer postoperative radiation to the preserved breast along with hormone therapy only. There was no delay in postoperative wound healing, and the patient was free of metastatic recurrence for 9 months after surgery.

**Conclusion:**

We performed surgery, postoperative radiotherapy, and hormonal therapy in a breast cancer patient with vascular EDS without major complications.

**Supplementary Information:**

The online version contains supplementary material available at 10.1186/s40792-024-01997-5.

## Background

Ehlers–Danlos syndrome (EDS) is an inherited connective tissue disease characterized by hyperextensibility of the skin and joints and tissue fragility of the skin and blood vessels due to abnormal collagen genes. It is classified into 13 types. EDS occurs in approximately 1 in 5,000 people, with vascular EDS being the most common type, as it is caused by type III collagen, which is the most severe form, with a high risk of arterial dissection, aneurysm rupture, and intestinal rupture [1–3].

However, there are few reports of breast cancer in patients with EDS and none in Japan. Herein, we report an extremely rare case of breast cancer in a patient with vascular EDS, along with literature review.

### Case presentation

The patient was a 46-year-old woman who, at the age of 20 years, was suspected of having EDS after a hospital visit when subcutaneous hemorrhage appeared in her lower leg after prolonged sitting. At the age of 35 years, when she became pregnant, a genetic diagnosis was made, and a *COL3A1* gene mutation was confirmed. There was no family history of genetically diagnosed EDS in the patient's family at present. Genetic counselling is continuing for the family, and we are considering whether a confirmatory test should be performed. And there was no family history that would raise suspicion of hereditary breast–ovarian cancer syndrome. The patient was referred to our department in July 2023 for further examination and treatment.

**Fig. 1:**
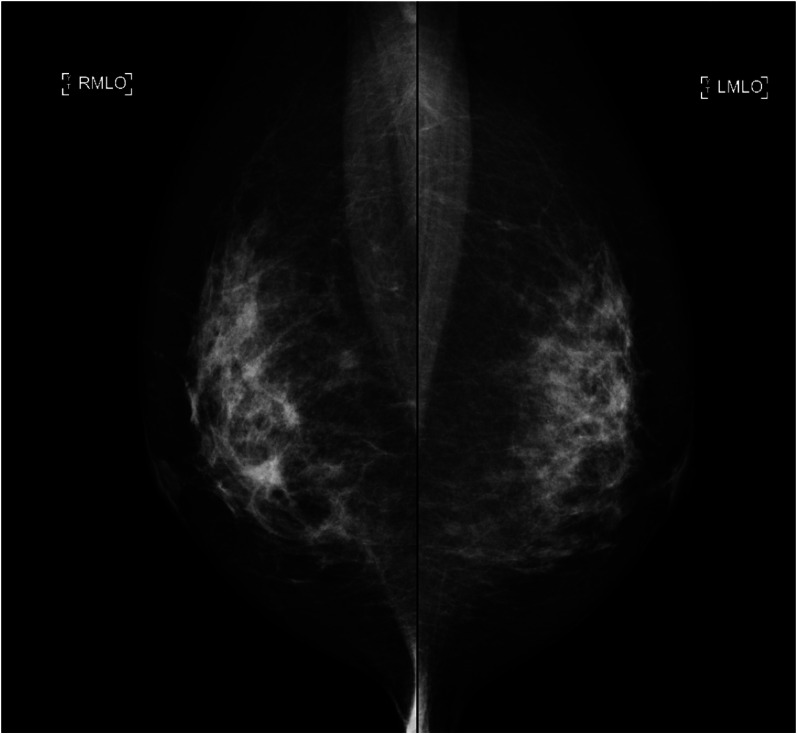
Mammography revealed no obvious bilateral abnormalities (Category 1)

Palpation revealed an internal hemorrhage in the left lower outer region of the left breast. No mass was palpable. Thin translucent skin was also observed. Mammography revealed no obvious bilateral abnormalities (Category 1) (Fig. 1).

**Fig. 2:**
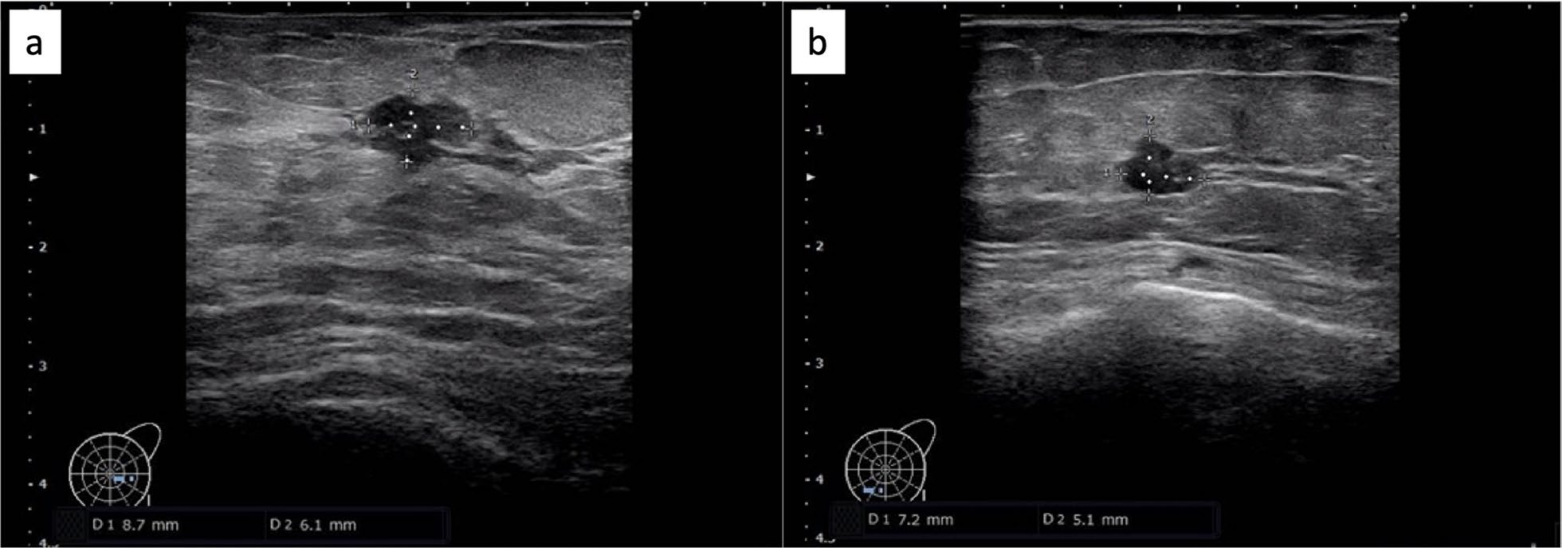
Breast ultrasound findings. **a** 4 × 3 mm oval, well-defined, smooth, hypoechoic mass in the left lower outer region of the left breast (Category 3). **b** 6 × 6 mm round, well-defined, smooth, hypoechoic mass in the left lower inner region of the left breast (Category 4)

Breast ultrasonography revealed an oval, well-defined, smooth, hypoechoic mass measuring 4 × 3 −mm in size in the left lower outer region of the left breast(left 5 o'clock position) (Category 3) (Fig. 2a). In addition, a 6 × 6 -mm large round, borderline clear, and smooth hypoechoic mass was found in the left lower inner region of the left breast (left 7 o'clock position) (Category 4). Considering the history of EDS, an additional cytological examination was performed for the left lower inner region, and the diagnosis was malignant (ductal carcinoma) (Fig. 2b).

**Fig. 3:**
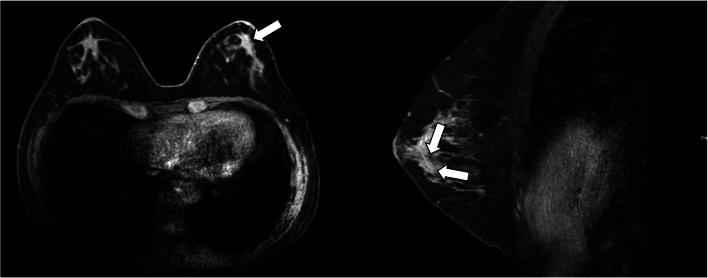
MRI (T2-weighted image) showed a clustered ring enhancement (arrow) in the left lower outer region of the left breast, approximately 9 mm in diameter, which was suspected to be DCIS

Contrast-enhanced mammography showed clustered ring enhancement of approximately 9 mm in the left lower outer region of the left breast, suggestive of DCIS (Fig. 3).

Whole-body PET–CT showed marked accumulation of SUVmax 3.4 in the D region of the left breast. There was also localized accumulation with SUVmax 4.2 in the gastroesophageal region.

After the above examination, a geneticist and gastroenterologist were consulted, and upper gastrointestinal endoscopy was performed for a more detailed examination of the stomach, taking care to avoid bleeding. The results showed reflux esophagitis with no obvious abnormalities.

Initially, total mastectomy was planned because of the two lesions in the left breast; however, considering the risk of bleeding and delayed wound healing, partial left mastectomy and sentinel lymph node biopsy were performed for the two lesions. In August 2023, partial left mastectomy and sentinel lymph node biopsies were performed under general anesthesia.

**Fig. 4:**
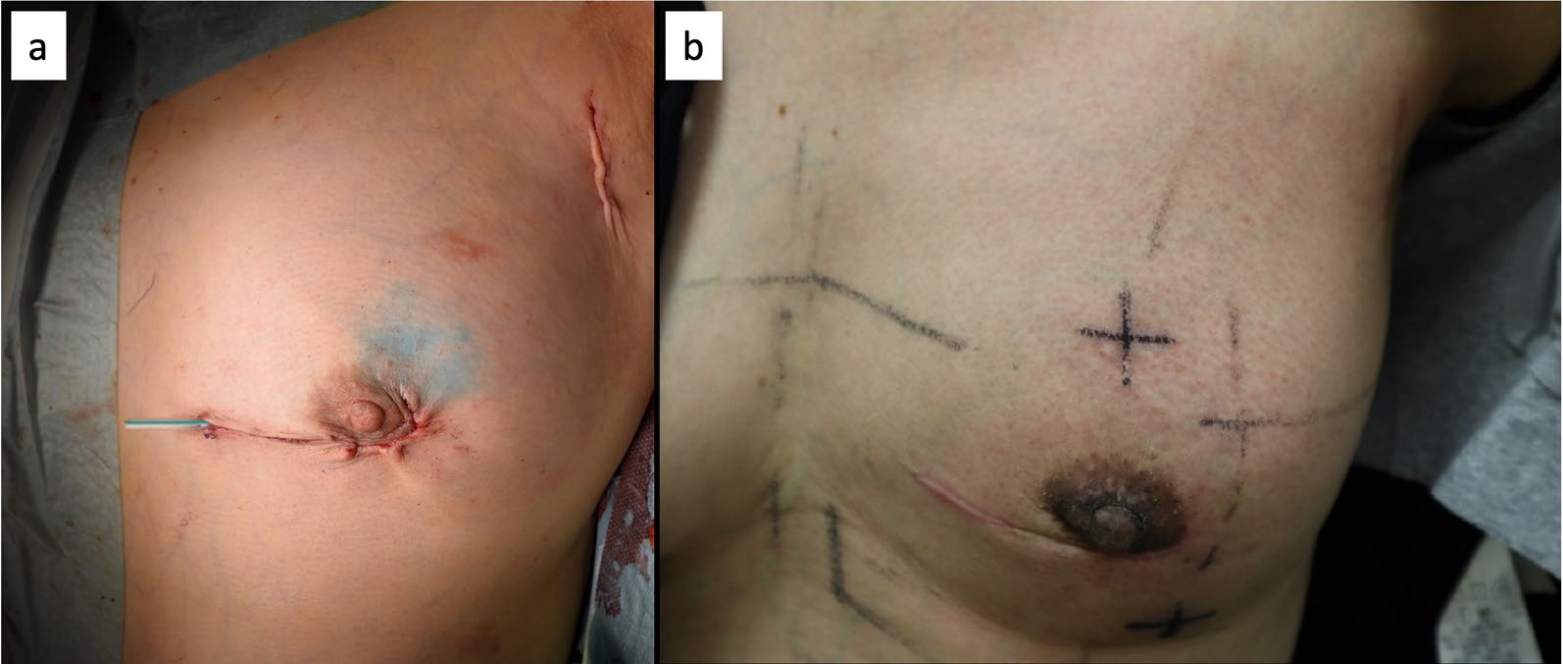
**a** Photographs taken immediately after surgery. Thin skin findings with high vascular permeability in the anterior thoracic region. **b** No delayed wound healing was observed at 3 months postoperatively

Sentinel lymph node biopsy was performed using a combination of the RI (99mTc phthalate) and dye (indigo carmine) methods, and one sentinel lymph node was identified and diagnosed as negative for metastases by rapid pathology. First, breast-conserving surgery was performed in the left lower inner region of the left breast. As atypical cells were found in one of the two margins on rapid pathology, additional resection was performed, and the margins were submitted again with a negative diagnosis on rapid pathology. Next, breast-conserving surgery was performed in the left lower outer region of the left breast, and four margins were submitted for rapid pathological diagnosis, all of which were negative. After confirming hemostasis, a Penrose drain was placed only in the breast-sparing area, and the operation was completed (Fig. 4a). Tissue fragility was observed intraoperatively; however, the operation was carefully performed, and the blood loss was approximately 75 g.

The patient had a good a favorable postoperative course. The drain was removed on postoperative day 3, and the patient was discharged on postoperative day 4.

At the outpatient visit on postoperative day 10, the subcutaneous hematoma in the wound was very mild and no swelling was observed. There was no lymphatic effusion. Although the effect of surgery is considered unlikely, two months after surgery, before the start of postoperative radiotherapy, a sudden hematoma developed after pain in the right axilla to the lateral thorax on the side contralateral to the side where the operation was performed. The patient was hospitalized for four days, and the bleeding resolved mildly with conservative treatment (Supplementary Fig. 1). There was no obvious delay in wound healing at 3 months postoperatively (Fig. 4b).

**Fig. 5:**
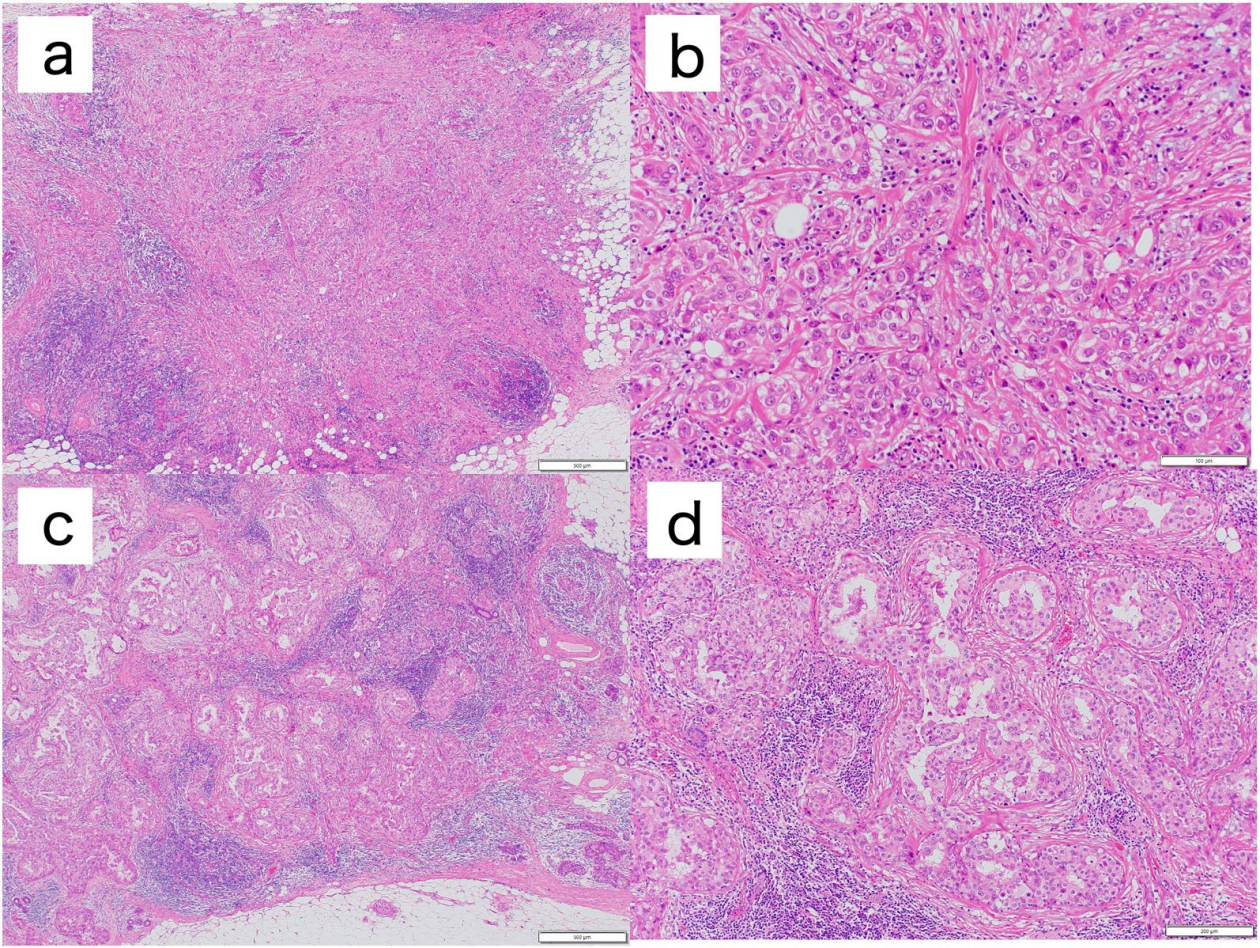
Histopathological findings (HE staining). **a**, **b** Lesion in the left lower inner region of the left breast was invasive ductal carcinoma. **c**, **d** Lesion in the left lower outer region of the left breast was non-invasive ductal carcinoma. Scale bar: 500 μm (**a**, **c**), 100 μm (**b**), 200 μm (**d**)

The histopathological findings were as follows. The lesion in the left lower inner region of the left breast was an invasive ductal carcinoma of the breast [enhanced type, pT1b, pN0, cM0, Stage IA, estrogen receptor (ER)-positive (95%), progesterone receptor (PR)-positive (< 1%), HER2 score 2 equivocal, HER2- FISH-positive, Ki-67 labeling index 18%] (Fig. 5a).

The lesion in the left lower outer region of the left breast was a non-invasive ductal carcinoma of the breast [pTis, pN0, cM0, pStage 0, ER-positive (70%), PR-positive (25%), HER2-positive (score3),and Ki-67 labeling index 18%] (Fig. 5b).

**Fig. 6:**
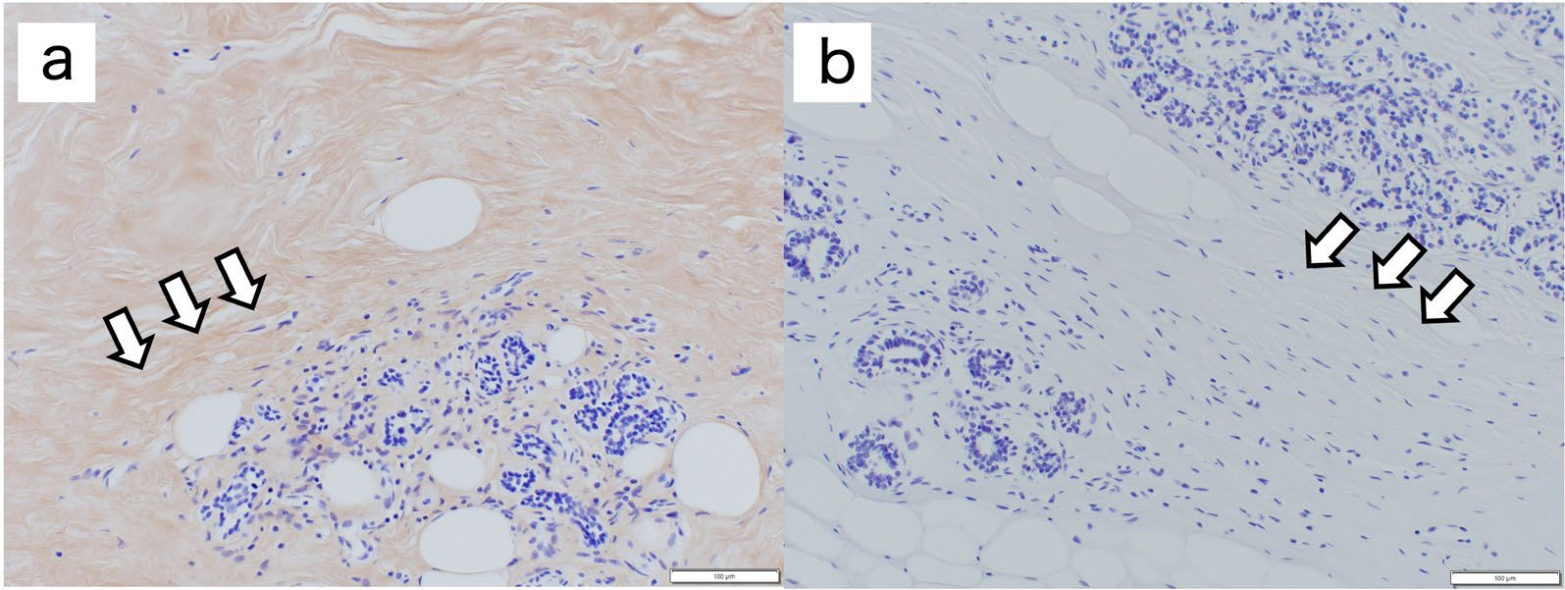
Histopathological findings (type III collagen immunostaining). The mammary gland tissue of a woman of the same generation (**a**) was positive, but the mammary gland tissue of this case (**b**) was negative. Scale bar: 100μm (**a**, **b**)

Immunostaining for type III collagen showed no positive fibers in the mammary tissue surrounding the tumor (Fig. 6b) [reference finding: positive findings were observed in the mammary tissue of a woman of the same age (Fig. 6a), as in the control case]. The histopathological findings did not suggest any obvious abnormal vascular wall structures or fragility.

The postoperative pathology results showed that with a diagnosis of Luminal HER2-type, the 10-year breast cancer mortality risk using a prognostic tool for early-stage breast cancer “Predict” (https://breast.predict.nhs.uk) was 5%, while endocrine therapy + chemotherapy + anti-HER2 therapy resulted in a 1% reduction in mortality risk. Endocrine therapy (tamoxifen), chemotherapy, and anti-HER2 therapy (taxanes + trastuzumab) are recommended as postoperative adjuvant therapies. However, the patient's 6 mm invasion diameter and histological grade I suggested a low-grade malignancy, and the patient herself wanted to avoid chemotherapy + anti-HER2 therapy if possible. Therefore, postoperative radiotherapy was initiated (42.4 Gy/16 irradiations to the preserved part of the left breast from October to November 2023).

Because HER2 had a score of 2, BluePrint, a multigene assay, was used as an adjunct to determine the treatment strategy. The results showed that the BluePrint molecular subtype was also HER2-type. The final decision was based on the patient's willingness and prognosis, as predicted by Predict. Predict is a prognostic tool for early-stage breast cancer created using the UK Cancer Registry database. By entering the diameter of tumor invasion, presence and number of axillary lymph nodes, grade, age, menopausal status, ER status, HER2 status and Ki67, the survival rate (baseline risk) when surgery alone is performed). And the patient was administered oral antiestrogen therapy for 10 years as postoperative treatment.

At the time of this report, 9 months have passed since the surgery, and the patient remains an outpatient without metastatic recurrence. The Vascular Surgery Department will also conduct follow-up EDS with CT scans every 6 months.

## Discussion

EDS is characterized by hyperextensibility of the skin and joints and easy bleeding associated with fragility of the skin and blood vessels. A new international classification system was proposed in 2017; therefore, 13 different disease types are currently used [3].

Vascular EDS, an autosomal dominant genetic disorder caused by mutations in the type III collagen gene (*COL3A1*), is estimated to occur in approximately 5% of all patients with EDS (approximately 1 in 50,000–200,000), with 77% reported to have serious complications such as arterial rupture, dissection, or aneurysm [4, 5].

The type III collagen gene (*COL3A1*), the causative gene of vascular EDS, is distributed in many important organs that require stretching, including the heart, blood vessels, gastrointestinal tract, and uterus. This genetic mutation makes it a more severe form of the disease than other forms [6]

Furthermore, it has been reported that 25% of patients develop these serious complications by the age of 20 years and 80% by the age of 40 years, with a median age of death of 48 years [1].

Several complications are associated with surgery and other invasive procedures. Standby surgery should be avoided, and a certain view of the treatment has not yet been reached.

Treatment of early-stage breast cancer may be preceded by surgical or drug therapy; however, at present, local treatment with surgical therapy is mandatory, except in limited cases such as low-grade, non-invasive cancer [7].

The patient had two lesions, one of which was very early-stage breast cancer (non-invasive ductal carcinoma, cStage 0), and the other had not yet been histologically diagnosed. Therefore, we decided that prior surgical treatment was desirable for both diagnosis and treatment. The patient was judged to be amenable to general anesthesia after preoperative discussions with the attending vascular surgeon and anesthetist at the hospital. During the operation, the patient's blood pressure, pulse, and other circulatory parameters were stable without major problems, and intraoperative findings showed tissue fragility during surgical procedures, such as dissection and resection. No abnormal bleeding was observed, and the surgery was completed without complications. No postoperative hemorrhage as a complication, wound infection, or obvious delayed wound healing was observed. Irradiation of the left breast was performed as planned and completed without any problems.

Regarding postoperative radiotherapy, postoperative irradiation after partial mastectomy in patients with early-stage breast cancer is strongly recommended by various guidelines. However, there is little evidence regarding the safety of radiotherapy in patients with EDS. For patients with breast cancer and EDS, Hsieh et al. reported that no significant acute or late toxicity was observed after conventional or hypofractionated radiotherapy of the preserved breast, with only grade 1 dermatitis in all patients (four) [8]. In contrast, Chau et al. reported that postoperative radiotherapy with additional boost irradiation in a patient with breast cancer with vascular EDS, as in the present case, resulted in grade 3 rather than severe dermatitis after 3 weeks [9]. Based on these reports, we judged that breast radiotherapy was likely to be safe and performed postoperative radiotherapy in this case because the patient had negative margins and did not require "additional boost irradiation”. As a result, no post-irradiation complications have been observed.

## Conclusions

We encountered a patient with early-stage breast cancer and rare vascular EDS who underwent surgery under general anesthesia with preoperative treatment planning in collaboration with various departments. Postoperative radiotherapy was successfully completed without major adverse events. In conclusion, in patients with vascular EDS with high comorbidities requiring careful management, surgery and postoperative radiotherapy may be acceptable with careful planning and manipulation.

### Supplementary Information

Additional file 1: figure 1.Subcutaneous hemorrhage in the axillary to lateral thoracic region on the side contralateral to the operation occurred 2 months after surgery.

## Data Availability

No datasets were created and analyzed in this study; therefore, no data were made publicly available.

## Abbreviations

HER2: Human epidermal growth factor receptor 2
FISH: Fluorescence in situ hybridization
COL3A1: Collagen type III alpha 1 chain
MRI: Magnetic resonance imaging
PET–CT: Positron emission tomography–computed tomography
