# Supplementary figures and images for: Surgical and irradiated case of early breast cancer in a patient with Ehlers–Danlos syndrome
Source: Surg Case Rep. 2024 Aug 23;10:195. doi: 10.1186/s40792-024-01997-5 (PMC11343928; doi:10.1186/s40792-024-01997-5)

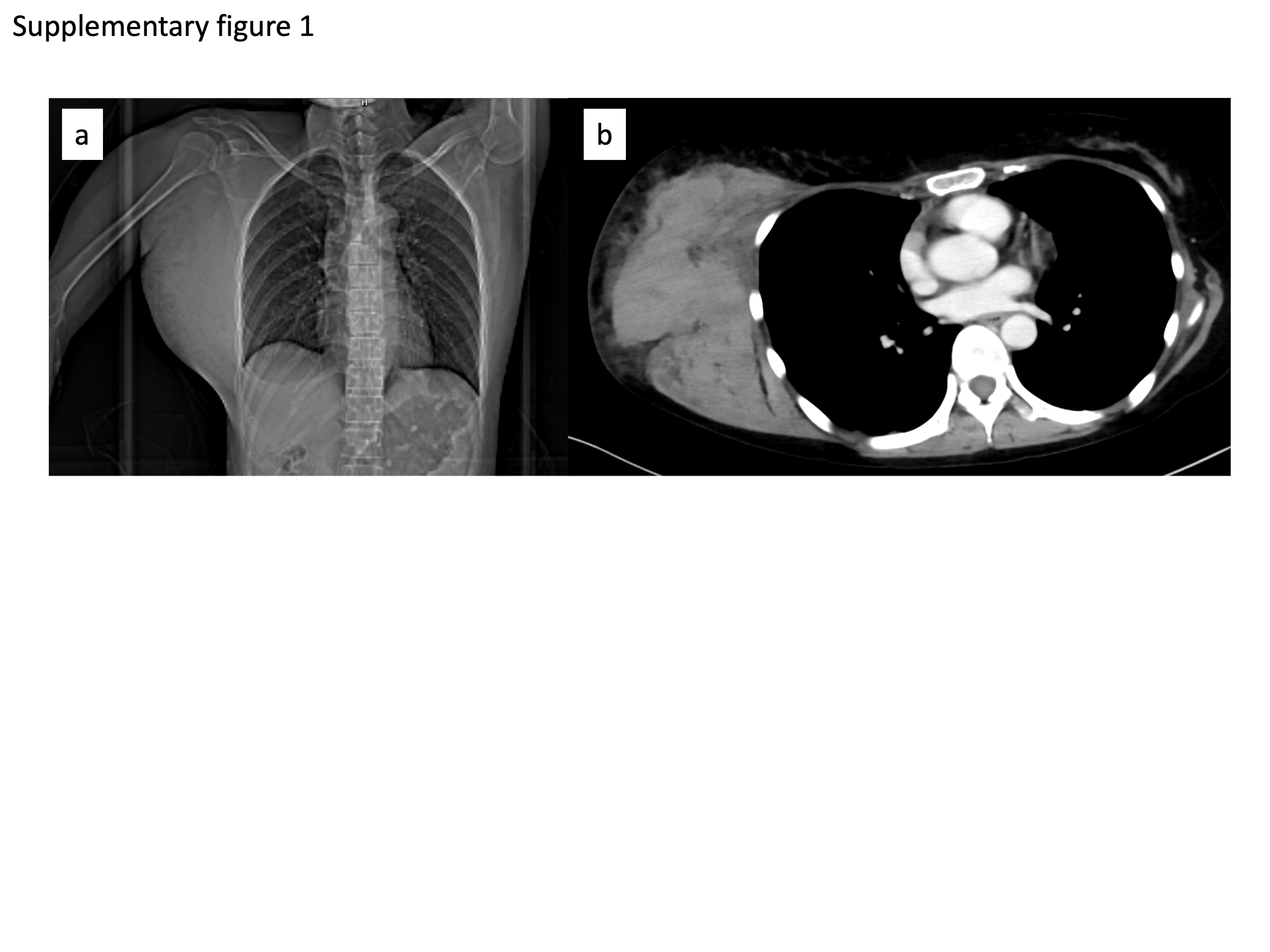

Supplement: Supplementary file 1 — Additional file 1: figure 1.Subcutaneous hemorrhage in the axillary to lateral thoracic region on the side contralateral to the operation occurred 2 months after surgery. [file 40792_2024_1997_MOESM1_ESM.tiff]
